# NO-Responsive Oleanolic Acid Self-Assembled Micelles Co-Loaded with BAY 11-7082 for Synergistic Chondroprotection and Anti-Osteoarthritis Therapy

**DOI:** 10.3390/bioengineering13080908

**Published:** 2026-08-11

**Authors:** Dandan Zhang, Zhigang Zhang, Dingxing Huang, Zhuoran Sun, Jiamin Huang, Chi Zhang, Qingyang Zeng, Qiling Liu, Wenzhuo Chen

**Affiliations:** 1 School of Public Health, Shaanxi University of Chinese Medicine, Xianyang 712046, China1261043@sntcm.edu.cn (J.H.); 523100401432@email.sntcm.edu.cn (Q.Z.); 2 School of Clinical Medicine, Shaanxi University of Chinese Medicine, Xianyang 712046, China; 523030107519@email.sntcm.edu.cn; 3 Xianyang Central Hospital, Xianyang 712000, China; 4 Key Laboratory of Functional Textile Material and Product, Ministry of Education, School of Environment and Chemical Engineering, Xi’an Polytechnic University, Xi’an 710048, China

**Keywords:** oleanolic acid, nitric oxide responsiveness, NF-κB inhibitor, chondrocyte protection, osteoarthritis, supramolecular drug delivery

## Abstract

Osteoarthritis is an irreversible degenerative joint disease driven by sustained NF-κB-mediated inflammatory responses, and conventional intra-articular hyaluronic acid or small-molecule NF-κB inhibitors cannot achieve targeted on-demand treatment due to poor solubility, rapid clearance and lack of lesion microenvironment responsiveness. OA with inherent anti-chondrolytic activity can self-assemble into nanocarriers in water, yet it lacks stimuli-responsive capacity. Herein, we rationally designed and synthesized an OA-Der by covalently conjugating o-phenylenediamine fragments to the OA backbone. ^1^H NMR and HRESI-MS spectra fully verified the accurate chemical structures of intermediate and final OA-Der. Blank OA-Der micelles exhibited uniform spherical core–shell nanostructures (50–150 nm) under TEM and AFM, while pathological high NO triggered complete disassembly of micellar assemblies. We further co-assembled OA-Der with NF-κB inhibitor BAY 11-7082 to construct NO-responsive BAY@OA-Der supramolecular micelles. In vitro experiments using human C28/I2 chondrocytes with LPS-induced inflammatory injury demonstrated that BAY@OA-Der significantly improved cell viability and reduced apoptotic chondrocyte proportion. At mRNA and protein levels, the supramolecular micelle formulation remarkably suppressed NF-κB p65 phosphorylation, downregulated cartilage-degrading ADAMTS5, and upregulated ACAN compared with free BAY or blank OA-Der. Collectively, this natural bioactive self-assembled NO-responsive delivery platform achieves synergistic anti-inflammatory and matrix-protective effects by precisely releasing drugs at NO-overexpressed osteoarthritis inflammatory sites and offers an in vitro design strategy for osteoarthritis responsive delivery systems.

## 1. Introduction

Osteoarthritis is a prevalent degenerative joint disease worldwide [1]. Currently, there is no curative treatment for this disorder, and conventional symptomatic therapies cannot halt disease progression [2]. Chronic inflammation, predominantly driven by the excessive activation of the nuclear factor-κB (NF-κB) signaling pathway [3], is fundamentally recognized as the primary cause of progressive articular cartilage degradation [4].

While targeting the NF-κB signaling pathway is highly promising [5,6], conventional small-molecule NF-κB inhibitors exhibit limited therapeutic efficacy in clinical osteoarthritis treatment [7]. This is mainly due to their poor water solubility, short retention time in joint cavities, and the absence of tissue targeting [8,9]. To overcome these barriers, nanocarriers are often employed [10,11]; however, most existing delivery systems are artificially synthesized inert materials [12]. Although they can improve pharmacokinetic profiles, they possess no intrinsic biological activity, which restricts their comprehensive therapeutic potential. Hydrogels prolong intra-articular retention via depot formation, yet they are limited to passive release with poor cartilage penetration and no inflammatory responsiveness [13]. Liposomes have good biocompatibility but suffer from low drug loading, poor synovial stability and no intrinsic activity [14]. Synthetic polymeric micelles realize adjustable encapsulation, yet their polymer backbones remain limited by biodegradability and immunogenicity [15]. Carrier-free self-assembled nanomedicines are assembled from bioactive molecules without exogenous polymers, acting as both carrier and therapeutic agent to avoid carrier-associated risks while providing low cytotoxicity, high cellular uptake and sustained drug release for synergistic anti-inflammatory and chondroprotective effects [16]. BAY 11-7082 acts as a potent NF-κB inhibitor, yet its strong hydrophobicity and inferior water solubility drastically reduce bioavailability upon intra-articular injection, and unregulated NF-κB inhibition may disrupt immune homeostasis [17,18]. Therefore, developing novel functional drug delivery platforms that possess both robust loading capacity and inherent bioactivity is urgently needed to precisely deliver NF-κB inhibitors for osteoarthritis treatment [19].

Oleanolic acid (OA), a natural pentacyclic triterpenoid with inherent anti-inflammatory and chondroprotective activities, has attracted extensive attention in recent years [20]. A distinct advantage of this molecule lies in its capacity to spontaneously form uniform, stable supramolecular micelles via self-assembly in aqueous media through highly dynamic intermolecular non-covalent interactions such as hydrophobic interactions, hydrogen interactions, and π-π interactions, rendering it an ideal candidate for constructing advanced drug carriers [21,22,23]. Although reactive oxygen species (ROS), acidic pH and matrix metalloproteinases (MMPs) are upregulated in osteoarthritis, nitric oxide (NO) was adopted as the trigger; it is a key driver of cartilage degradation, and its large concentration difference between healthy and inflamed joint tissue enables targeted on-demand drug release at inflammatory sites [24,25]. On the other side, the osteoarthritic microenvironment is characterized by pathologically elevated levels of NO [26]. Rather than merely acting as an inflammatory mediator, this elevated NO serves as a promising endogenous trigger [27]. Through rational molecular design, the self-assembled OA scaffold can be engineered to respond to this NO stimulus, thereby achieving on-demand, controlled drug release specifically at inflammatory lesions of osteoarthritis.

Driven by this rationale, we chemically engineered the OA skeleton by introducing NO-responsive functional groups to synthesize a novel NO-responsive supramolecular derivative (OA-Der). The obtained OA-Der fully retained the excellent self-assembly properties of the parent compound and readily formed uniform supramolecular micelles in water [28]. Crucially, triggered by high-NO conditions mimicking the inflammatory microenvironment of osteoarthritis, these OA-Der micelles undergo dynamic supramolecular disassembly to realize precise on-site drug release. Using this natural bioactive nanoplatform, we further encapsulated the NF-κB inhibitor BAY 11-7082 to construct NO-responsive drug-loaded supramolecular micelles (BAY@OA-Der) via a co-assembly strategy. In vitro cellular assays verified that BAY@OA-Der could specifically target inflammatory microenvironments, synergistically inhibit the NF-κB pathway, alleviate chondrocyte damage, and restore cartilage matrix metabolic homeostasis. This research demonstrates the great potential of OA-based self-assembled nanostructures as highly functional drug carriers, providing a promising supramolecular strategy for constructing inflammation-responsive delivery systems against osteoarthritis in vitro.

## 2. Materials and Methods

### 2.1. Chemicals and Instruments

All chemicals were purchased from commercial sources. The ^1^H and ^13^C NMR spectra were acquired on a Bruker AVANCE III 400 MHz magnetic resonance spectrometer (Bruker Corporation, Woltzbach, Germany) in deuterated chloroform (CDCl_3_) or deuterated dimethyl sulfoxide (DMSO-d_6_). Data for NMR spectra are listed as follows: chemical shifts are reported as δ in units of parts per million (ppm) relative to chloroform-d (δ 7.26, s); multiplicities are reported as follows: s (singlet), d (doublet), t (triplet), q (quartet), dd (doublet of doublets), m (multiplet) and br (broadened); coupling constants are reported as a J value in Hertz (Hz); the number of protons (n) for a given resonance is indicated as nH and is based on the spectral integration values. Ultraviolet–Visible Spectroscopy (UV-Vis) spectra were measured by using a Shimadzu UV-2550 model spectrophotometer (Shimadzu Corporation, Kyoto, Japan). IR spectra were measured using a TENSOR27 spectrometer (Bruker Corporation, Woltzbach, Germany) and are reported in cm^−1^. Hydrodynamic diameter was measured by using a Zeta sizer Nano ZS90 (Dandong Bettersize Instruments Ltd., Dandong, China) before the sample was filtered. The sample for transmission electron microscopy (TEM, ThermoFisher Scientific, Waltham, MA, USA) and high-angle annular dark field-scanning transmission electron microscopy (HAADF-STEM, Keyence, Osaka, Japan) was examined by a FEI Talos F200X instrument (ThermoFisher Scientific, Waltham, MA, USA). Atomic force microscope (AFM, Quantum Design, Beijing, China) was recorded in tapping mode under ambient conditions by using a Bruker Dimension FastScan (Bruker Corporation, Woltzbach, Germany) and Dimension Icon instrument (Bruker Corporation, Woltzbach, Germany).

For cell experiments, fetal bovine serum (FBS, G8033, Servicebio Technology, Wuhan, China), penicillin–streptomycin (G4015, Servicebio Technology, Wuhan, China), and Dulbecco’s Modified Eagle Medium/Ham’s F-12 Nutrient Mixture (DMEM/F12, G4502, Servicebio Technology, Wuhan, China) medium were obtained from standard vendors. Lipopolysaccharide (LPS, GC205009, Servicebio Technology, Wuhan, China) was used to establish the in vitro inflammatory model.

### 2.2. Synthesis and Characterization of the OA-Der

Firstly, the NO-responsive intermediate was synthesized. Briefly, Boc-protected 4-bromo-2-nitroaniline (B122481, Aladdin Biochemical Technology, Shanghai, China) was reduced by sodium dithionite (S433997, Aladdin Biochemical Technology, Shanghai, China), followed by an azidation reaction to introduce azide groups. In this study, naturally occurring oleanolic acid with intrinsic aqueous self-assembly property was used as the parent molecule. An amidation reaction was carried out between oleanolic acid and the as-prepared NO-responsive intermediate, catalyzed by 4-dimethylaminopyridine (DMAP, D109207, Aladdin Biochemical Technology, Shanghai, China) and pyridine (P816288, Macklin Biochemical Technology, Shanghai, China). The crude product was purified by silica gel column chromatography to obtain the target NO-responsive derivative, designated as OA-Der.

The chemical structure of OA-Der was characterized by ^1^H NMR (Bruker AVANCE NEO 400M, DMSO-d_6_) and electrospray ionization mass spectrometry (ESI-MS, Thermo TSQ Quantum Access MAX, ThermoFisher Scientific, Waltham, MA, USA). The measured mass-to-charge ratio was compared with the theoretical molecular weight to confirm the successful synthesis of OA-Der. OA-Der was preserved under conventional storage conditions (−20 °C, protected from light).

### 2.3. Preparation and NO-Responsive Characterization of BAY@OA-Der Drug-Loaded Micelles

Benefiting from the inherent intermolecular non-covalent interactions and self-assembly capability of OA-Der, BAY@OA-Der drug-loaded micelles were fabricated via a one-pot co-assembly strategy. OA-Der and BAY 11-7082 were co-dissolved in organic solvent at a predetermined ratio. The mixed solution was slowly dropped into deionized water under magnetic stirring, and the co-assembly of drug and carrier was realized relying on the self-assembly characteristic of OA-Der. After homogenization, the organic solvent was removed by slow evaporation to acquire homogeneous BAY@OA-Der micelle dispersion.

TEM and AFM were employed to observe the morphology of BAY@OA-Der micelles. The morphological changes in micelles before and after NO treatment were recorded to evaluate their NO-triggered disassembly behavior.

### 2.4. Cell Culture and Group Treatment

Human chondrocyte line C28/I2 was cultured in DMEM/F12 medium supplemented with 10% FBS and 1% penicillin–streptomycin at 37 °C in a humidified atmosphere containing 5% CO_2_. To establish the in vitro osteoarthritis model, cells were stimulated with 10 μg/mL LPS. The following five groups were included: (1) control group: untreated chondrocytes; (2) LPS group: cells stimulated with 10 μg/mL LPS; (3) LPS + BAY group: LPS-stimulated cells treated with free BAY 11-7082; (4) LPS + OA-Der group: LPS-stimulated cells treated with blank OA-Der micelles; and (5) LPS + BAY@OA-Der group: LPS-stimulated cells treated with BAY-loaded OA-Der micelles.

### 2.5. Cell Viability Assay

Cell viability was evaluated using the Cell Counting Kit-8 (CCK-8) assay. Chondrocytes were seeded in 96-well plates (CCP-96H, Servicebio Technology, Wuhan, China) at 5 × 10^3^ cells/well and treated as described above. After incubation, 10 μL CCK-8 reagent (G4103, Servicebio Technology, Wuhan, China) was added to each well, and the plates were incubated for 2 h. The absorbance at 450 nm was measured using a microplate reader (ThermoFisher Scientific, Waltham, MA, USA), and cell viability was calculated relative to the control group.

### 2.6. Live/Dead Cell Staining

Chondrocytes were seeded in 6-well plates at a density of 8 × 10^4^ cells per well and incubated until reaching 80% confluence prior to various interventions. A Calcein-AM/PI double staining kit (G1707, Servicebio Technology, Wuhan, China) was applied to detect cell death. After treatment, the medium was aspirated, and cells were incubated with staining solution protected from light. Fluorescence microscopy was used to observe and image live cells with green signals and dead cells with red signals. Quantitative analysis of dead cell proportion was performed across several random fields.

### 2.7. Western Blot Analysis

Total cellular protein was extracted, and protein concentration was determined. Equal amounts of protein were separated by Sodium Dodecyl Sulfate-Polyacrylamide Gel Electrophoresis (SDS-PAGE, S665689, Aladdin Biochemical Technology, Shanghai, China) and transferred to Polyvinylidene Fluoride (PVDF, G6047, Servicebio Technology, Wuhan, China) membranes. Membranes were incubated with primary antibodies against aggrecan (ACAN, GB155687, Servicebio Technology, Wuhan, China), a disintegrin and metalloproteinase with thrombospondin motifs (ADAMTS5, GB150099, Servicebio Technology, Wuhan, China), phosphorylated p65 (p-p65), total p65, and beta-actin (β-actin) overnight at 4 °C, followed by incubation with secondary antibodies. Protein bands were visualized, and relative expression levels were quantified by densitometry.

### 2.8. Quantitative Real-Time PCR (qPCR)

Total RNA was extracted from chondrocytes, and cDNA was synthesized by reverse transcription. The mRNA expression levels of *ACAN* and *ADAMTS5* were analyzed by qPCR using the 2^−ΔΔCt^ method, with the housekeeping gene as an internal control.

### 2.9. Statistical Analysis

All experiments were performed at least three independent times. Data are expressed as mean ± SD. One-way ANOVA with Dunnett’s multiple comparisons test was adopted for statistical analysis. Comparisons were performed either between treatment groups and the control group, or between treatment groups and the LPS-induced model group in different sets of analyses. Differences with *p* < 0.05 were regarded as statistically significant.

## 3. Results

### 3.1. Synthesis and Structural Characterization of the OA-Der

**Figure 1:**
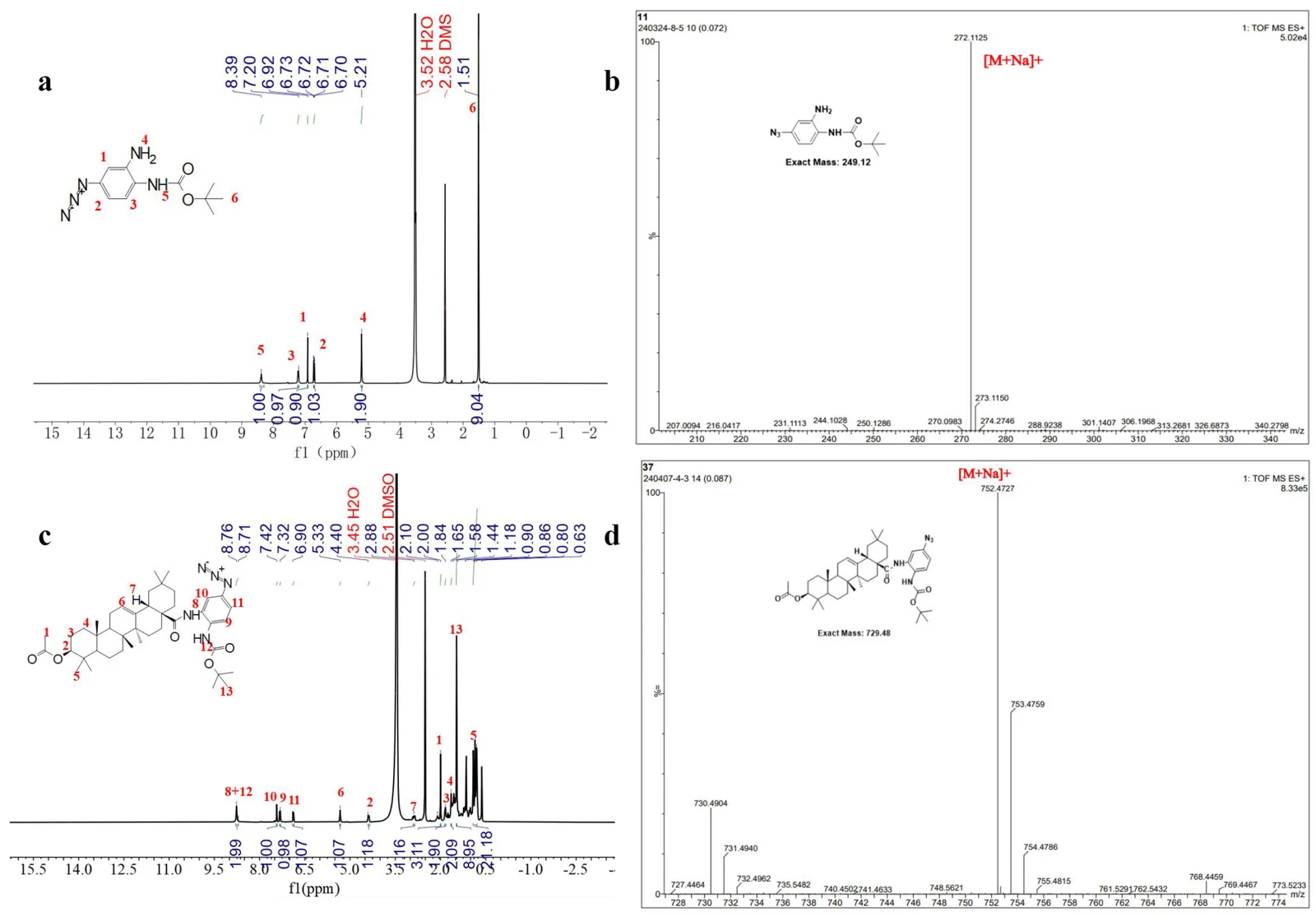
The synthesis of NO-Cleavable Azido–Aniline Intermediate and OA-Der: (**a**) ^1^H NMR spectrum (400 MHz, DMSO-d_6_, 298 K) recorded for NO-Cleavable Azido–Aniline Intermediate; (**b**) HRESI-MS for [M + Na]^+^. (**c**) ^1^H NMR spectrum (400 MHz, DMSO-d6, 298 K) recorded for OA-der; (**d**) HRESI-MS for [M + Na]^+^.

^1^H NMR and HRESI-MS were utilized to characterize the chemical structures of the NO-responsive intermediate and the target derivative OA-Der, with all corresponding spectra displayed in Figure 1. Figure 1a shows the ^1^H NMR spectrum (400 MHz, DMSO-d_6_, 298 K) of the NO-responsive o-phenylenediamine intermediate. Characteristic aromatic proton signals derived from azide-substituted aniline were observed, which matched the predicted molecular structure. The HRESI-MS spectrum of this intermediate is presented in Figure 1b, where a molecular ion peak at *m*/*z* = 272.1125 corresponding to the [M + Na]^+^ adduct was detected, consistent with the theoretical value.

For OA-Der, its ^1^H NMR spectrum (400 MHz, DMSO-d_6_, 298 K) is illustrated in Figure 1c. Distinctive proton signals originating from the native oleanolic acid skeleton and the conjugated NO-responsive functional moiety were simultaneously identified, verifying the successful conjugation of the two structural fragments. The HRESI-MS spectrum of OA-Der (Figure 1d) exhibited a molecular ion peak at *m*/*z* = 752.4759, which was in excellent accordance with the theoretical molecular weight of OA-Der. Collectively, these spectral characterizations confirm the successful synthesis of the NO-responsive derivative constructed based on the self-assembly skeleton of oleanolic acid.

### 3.2. Self-Assembly and NO-Responsive Disassembly of OA-Der Micelles

**Figure 2:**
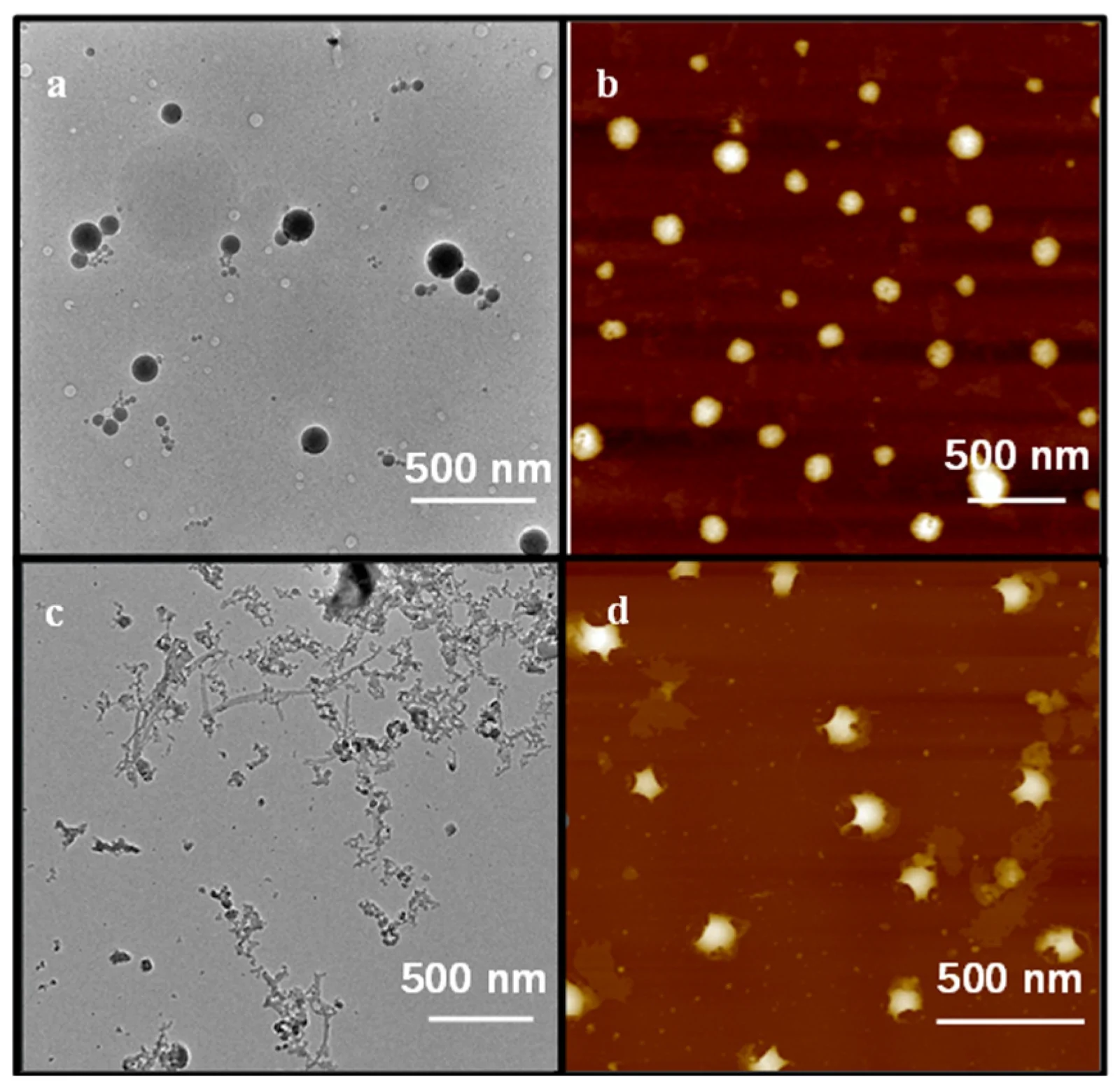
Formation of BAY@OA-Der and BAY@OA-Der after the treatment of NO. (**a**) TEM images of BAY@OA-Der; (**b**) AFM images of BAY@OA-Der; (**c**) TEM images of BAY@OA-Der after the treatment of NO. (**d**) AFM images of BAY@OA-Der after the treatment of NO.

Driven by intermolecular non-covalent interactions of the oleanolic acid scaffold, OA-Der can spontaneously and stably co-assemble with BAY 11-7082 to form supramolecular BAY@OA-Der micelles in aqueous medium. TEM and AFM were adopted to visualize the morphology of BAY@OA-Der micelles, as displayed in Figure 2a and Figure 2b, respectively. The TEM image (Figure 2a) and corresponding AFM image (Figure 2b) both revealed that BAY@OA-Der micelles were monodispersed uniform spherical nanoparticles with smooth surfaces and obvious core–shell architectures, which illustrated that chemical modification and co-loading of BAY barely impaired the intrinsic superior self-assembly capacity of oleanolic acid.

Upon incubation with NO, dramatic morphological transformation occurred to BAY@OA-Der micelles. As shown in Figure 2c (TEM) and Figure 2d (AFM), the intact spherical nanostructure of BAY@OA-Der micelles was completely disrupted, accompanied by the emergence of disordered debris and large aggregates. Such distinct morphological collapse validated that these drug-loaded oleanolic acid-derived micelles exhibited prominent NO-triggered disassembly behavior.

### 3.3. BAY@OA-Der Protects Chondrocyte Viability and Attenuates Cell Death in LPS-Induced Chondrocytes

**Figure 3:**
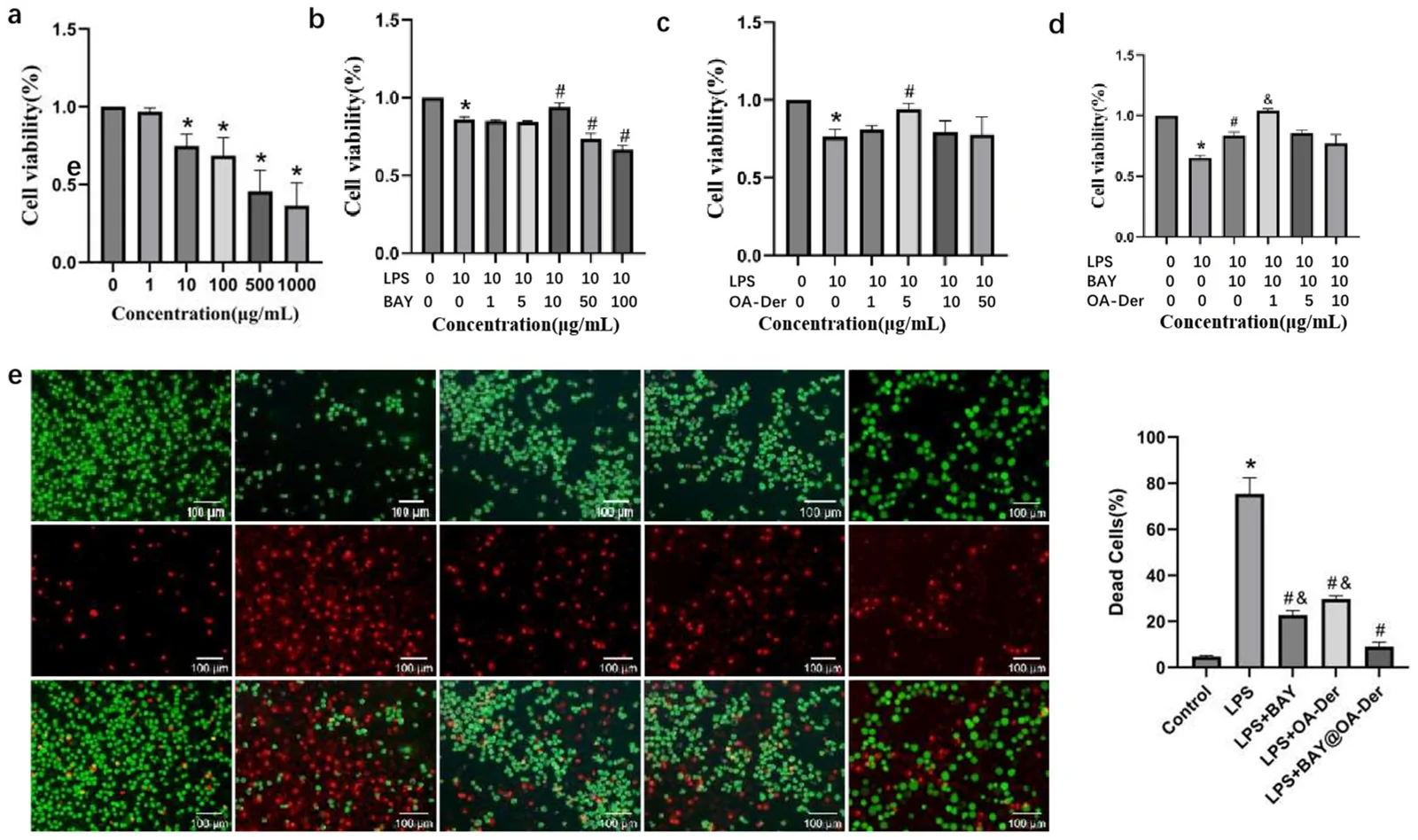
Cytoprotective effects of BAY@OA-Der against LPS-induced chondrocyte injury. (**a**) CCK-8 assay results showing dose-dependent cytotoxicity of LPS on chondrocytes; (**b**) cell viability of chondrocytes treated with gradient concentrations of free BAY 11-7082 under LPS stimulation; (**c**) cell viability of chondrocytes treated with gradient concentrations of blank OA-Der under LPS stimulation; (**d**) cell viability of chondrocytes co-treated with fixed-dose BAY and serial concentrations of OA-Der under LPS stimulation; (**e**) calcein-AM/PI live/dead double staining images and quantitative statistical histogram of dead cell proportion in each group (green fluorescence represents live cells; red fluorescence represents dead cells). Statistical annotations: * *p* < 0.05 versus the control group; # *p* < 0.05 versus the LPS group; and & *p* < 0.05 versus the LPS + BAY@OA-Der group.

The effects of LPS, free BAY 11-7082, OA-Der and the BAY@OA-Der formulation on chondrocyte viability were assessed via the CCK-8 assay, and the quantitative results are shown in Figure 3a–d. Figure 3a illustrated that LPS induced a dose-dependent reduction in cell viability, with significant cytotoxicity evident at concentrations of 10 μg/mL and higher (*p* < 0.05). Accordingly, 10 μg/mL LPS was selected to establish the in vitro osteoarthritis model for subsequent gradient screening experiments. Gradient treatment with free BAY was presented in Figure 3b, revealing that only 10 μg/mL BAY significantly restored the viability of injured chondrocytes; 1 and 5 μg/mL BAY failed to exert obvious improvement on cell viability, while high-dose BAY at 50 and 100 μg/mL exhibited prominent cytotoxicity, resulting in cell viability even lower than that of the LPS model group. Serial concentrations of blank OA-Der were also evaluated in Figure 3c, and only 5 μg/mL OA-Der markedly alleviated LPS-induced decline in cell viability, whereas no statistically significant protective effects were observed at 1, 10 and 50 μg/mL. Combined treatment with fixed 10 μg/mL BAY and different concentrations of OA-Der was summarized in Figure 3d, demonstrating that 1 μg/mL OA-Der produced synergistic effects with BAY to achieve the maximum cell viability, while co-administration with 5 or 10 μg/mL OA-Der provided no additional therapeutic benefits, with cell viability only slightly elevated compared with the LPS model group.

Calcein-AM/PI double staining was performed to directly observe chondrocyte survival and quantitatively calculate the proportion of dead cells, where green fluorescence indicated live cells and red fluorescence labeled dead cells (Figure 3e). Abundant red fluorescence and a sharply increased percentage of dead cells were observed in the LPS group. Separate interventions with free BAY or blank OA-Der both alleviated chondrocyte death to a certain extent. The BAY@OA-Der micelle group displayed the weakest red fluorescence and the lowest proportion of dead cells, with cellular survival levels comparable to the blank control group. Fluorescence images combined with quantitative data collectively verified that the co-assembled BAY@OA-Der supramolecular micelles possessed superior synergistic anti-apoptotic and chondroprotective capacities relative to free BAY or blank OA-Der alone, which could effectively relieve LPS-induced chondrocyte injury.

### 3.4. BAY@OA-Der Attenuates LPS-Induced Cartilage Matrix Degradation and NF-κB Signaling Activation

**Figure 4:**
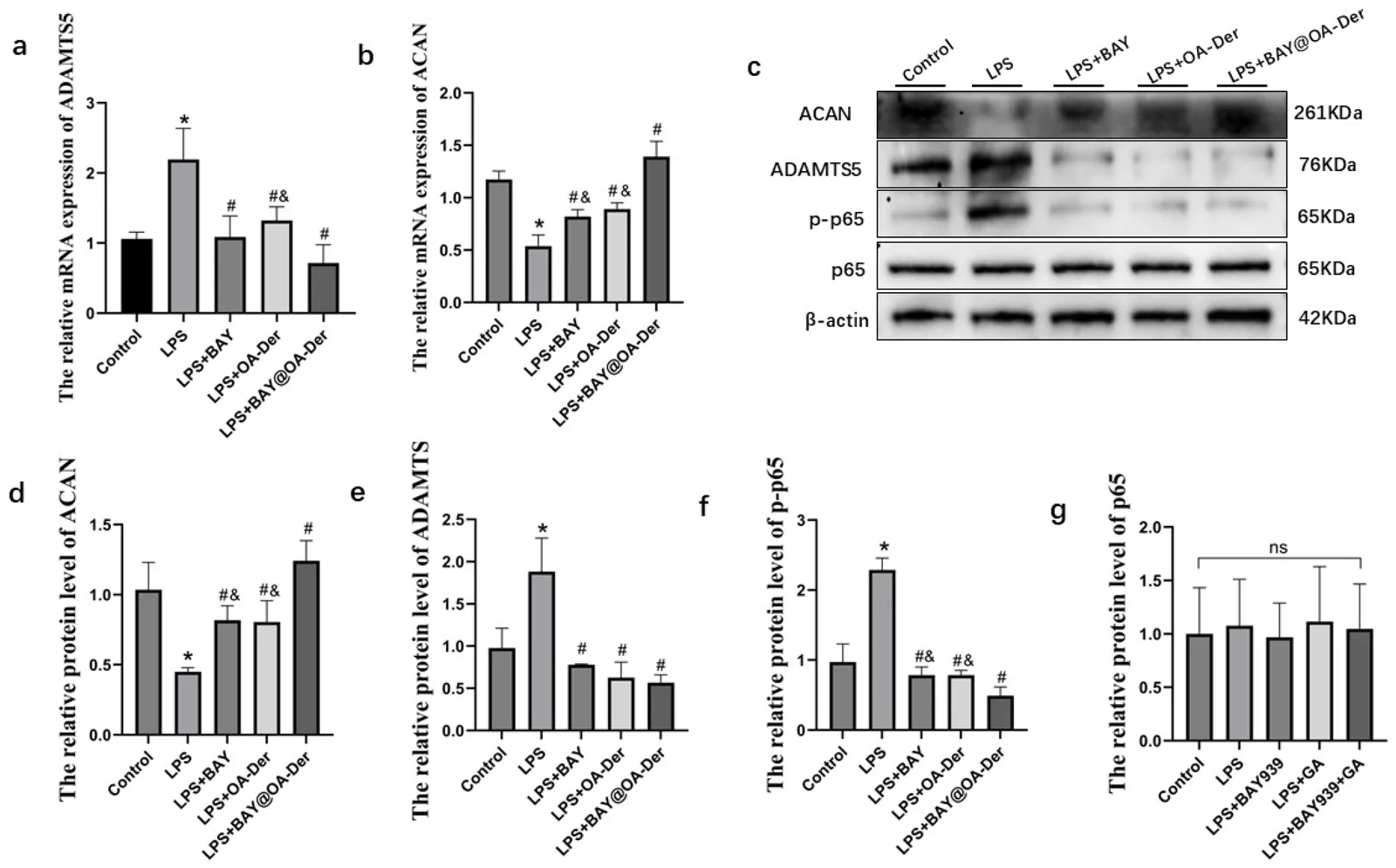
BAY@OA-Der suppresses LPS-triggered cartilage matrix degradation and NF-κB signaling overactivation in chondrocytes. (**a**,**b**) Relative mRNA expression levels of matrix catabolic gene ADAMTS5 and matrix anabolic gene ACAN detected by qRT-PCR; (**c**) representative Western blot bands of ACAN, ADAMTS5, phosphorylated p65 (p-p65) and total p65 protein, with β-actin as the internal reference; (**d**–**g**) quantitative statistical analysis of relative protein expression of ACAN, ADAMTS5, p-p65 and total p65. Statistical annotations: * *p* < 0.05 versus the control group; # *p* < 0.05 versus the LPS group; and & *p* < 0.05 versus the LPS + BAY@OA-Der group. “ns” denotes “not significant”.

The effects of LPS, free BAY 11-7082, OA-Der, and the BAY@OA-Der formulation on chondrocyte extracellular matrix metabolism and NF-κB pathway activation were assessed at both the mRNA and protein levels, and all related quantitative and blot results are presented in Figure 4a–g. At the transcriptional level, as shown in Figure 4a,b, LPS significantly upregulated the mRNA expression of the cartilage-degrading enzyme ADAMTS5 (*p* < 0.05) and downregulated the expression of the matrix component ACAN (*p* < 0.01), indicating the induction of chondrocyte catabolism and extracellular matrix degradation. Treatment with LPS + BAY or LPS + OA-Der partially reversed these changes, with both interventions reducing ADAMTS5 mRNA levels and increasing ACAN mRNA expression. Notably, the LPS + BAY@OA-Der group exhibited the most pronounced regulatory effect, showing a significant reduction in ADAMTS5 and a marked restoration of ACAN mRNA expression compared with the LPS group (*p* < 0.001).

Consistent with the qPCR results, Western blot bands are displayed in Figure 4c, and the corresponding quantitative protein data are summarized in Figure 4d–g. Western blot analysis revealed that LPS stimulation significantly decreased the protein level of ACAN and increased the protein expression of ADAMTS5, further confirming the disruption of extracellular matrix homeostasis. Additionally, LPS strongly activated the NF-κB pathway, as evidenced by a marked increase in p-p65 protein levels (Figure 4f) without a corresponding change in total p65 levels (Figure 4g). Treatment with LPS + BAY or LPS + OA-Der partially inhibited p65 phosphorylation and mitigated the LPS-induced alterations in ACAN and ADAMTS5 protein levels. Importantly, the LPS + BAY@OA-Der formulation exerted a more robust effect, effectively suppressing NF-κB activation, downregulating ADAMTS5, and restoring ACAN protein expression to levels significantly higher than those observed in the LPS + BAY or LPS + OA-Der groups. Collectively, these data demonstrate that the BAY@OA-Der formulation is superior to free BAY 11-7082 or OA-Der alone in inhibiting NF-κB pathway activation, preventing extracellular matrix degradation, and maintaining chondrocyte anabolic/catabolic balance under inflammatory conditions.

Additional supporting characterization data including ^13^C NMR spectra, cytotoxicity test, pro-inflammatory cytokine quantification and time-dependent UV-vis spectra are available in the Supplementary Materials (Figures S1–S5).

## 4. Discussion

To realize the precise design of our microenvironment-responsive supramolecular delivery system, the NO-cleavable intermediate and the target supramolecular building block (OA-Der) were rationally synthesized [29]. Their chemical structures were unambiguously elucidated via ^1^H NMR spectroscopy and ESI-MS. For the NO-responsive intermediate, the ^1^H NMR spectrum exhibited characteristic aromatic proton resonances corresponding to the azide-substituted aniline moiety, confirming the successful introduction of the NO-recognition site. Its ESI-MS spectrum displayed a distinct molecular ion peak at *m*/*z* = 272.1125, which perfectly matched the theoretical value of the [M + Na]^+^ adduct.

Subsequently, this NO-responsive moiety was covalently conjugated to the natural oleanolic acid skeleton. In the ^1^H NMR spectrum of the final OA-Der, the coexistence of the characteristic rigid cyclic proton signals derived from the parent oleanolic acid and the aromatic proton signals from the conjugated NO-responsive moiety provided solid evidence for the successful integration of the two functional fragments. Furthermore, the ESI-MS analysis of OA-Der presented a high-resolution molecular ion peak at *m*/*z* = 752.4759, which was in excellent agreement with its theoretical molecular weight. These spectral analyses collectively confirm the successful synthesis of the target NO-responsive derivative, laying a defined molecular foundation for the subsequent supramolecular engineering. This design cleverly combines the inherent bioactivity of natural molecules, natural self-assembly characteristics and microenvironment responsiveness, effectively overcoming the defects of traditional drug delivery systems, and provides a new in vitro research direction for developing functional nanocarriers for osteoarthritis intervention [30].

With the rationally designed chemical structure verified at the molecular level, we further investigated the supramolecular co-assembly behavior of this delivery system. To fabricate the functional drug-loaded nanomedicine, the hydrophobic NF-κB inhibitor BAY 11-7082 was introduced into the system during the aqueous self-assembly process. Driven by the highly dynamic intermolecular non-covalent interactions (predominantly hydrophobic forces and van der Waals interactions) inherent to the oleanolic acid skeleton [31], the amphiphilic OA-Der molecules and the hydrophobic BAY 11-7082 spontaneously co-assembled into stable [32], drug-loaded supramolecular micelles (BAY@OA-Der) in aqueous media.

Morphological characterizations were subsequently performed to visually confirm the successful formation of these supramolecular architectures. Both TEM and AFM results corroborated each other, demonstrating that the BAY@OA-Der co-assemblies were uniformly dispersed as regular spherical nanoparticles with distinct core–shell topologies and smooth surfaces. Crucially, the particle sizes observed in both TEM and AFM imaging were in excellent agreement, consistently distributed within a relatively narrow and well-defined range of 50–150 nm. This optimal nanoscale dimension not only confirms the robust co-assembly capability of the engineered oleanolic acid backbone but also falls within the ideal size window for enhancing cellular uptake and prolonging retention in joint cavities [33,34].

However, the stability of these supramolecular architectures is highly dependent on the microenvironmental stimuli. Since NO is abnormally accumulated in osteoarthritis inflammatory lesions and acts as a key inflammatory mediator [26], the NO-triggered structural disintegration of micelles can achieve on-demand drug release at lesion sites, reduce off-target drug release in normal tissues, and potentially lower systemic side effects. Upon exposure to pathological lesions accompanied by elevated endogenous NO levels that mimic the microenvironment of osteoarthritic lesions, the specific cleavage of the responsive functional groups abruptly disrupted the supramolecular amphiphilic balance of the building blocks. Consequently, the regular spherical architectures of the BAY@OA-Der micelles (50–150 nm) were thoroughly destroyed, rapidly transforming into irregular fragments and amorphous aggregates. This drastic morphological transition visually and unequivocally confirms the excellent NO-responsive dynamic disassembly performance of the co-assembled nanoplatform, enabling targeted drug release under a simulated inflammatory microenvironment in vitro. The compound self-assembles into spherical nanoparticles in water via synergistic non-covalent forces. Its bulky pentacyclic triterpene skeleton provides strong hydrophobic moieties that drive spontaneous aggregation to minimize interfacial energy and form spherical nanospheres, while intermolecular hydrogen bonds and π–π stacking from aromatic rings further stabilize the assemblies. These nanospheres undergo selective disassembly upon NO stimulation: NO reacts with azide-substituted aryl units to cleave amide bonds, breaking the molecular backbone and disrupting the hydrophilic–hydrophobic balance and hydrogen-bond network. Such molecular fragmentation leads to macroscopic collapse of spherical micelles under electron microscopy, enabling rapid on-site drug release. Similar NO-triggered cleavage and disassembly behavior of supramolecular architectures induced by hydrolytic reaction has been systematically illustrated in our previous work [35].

In vitro cellular experiments demonstrated that the BAY@OA-Der co-assembled micelles exerted superior chondroprotective and anti-inflammatory effects compared with free BAY 11-7082 and blank OA-Der micelles. On the one hand, the nanoscale size of micelles improves the solubility and cellular uptake efficiency of BAY 11-7082, enhancing its bioavailability [36]. On the other hand, the OA skeleton itself can inhibit inflammatory responses and protect chondrocytes [37]. The dual effects of the carrier and loaded drug jointly suppress the overactivation of the NF-κB pathway. As a core inflammatory signaling pathway, sustained NF-κB activation will upregulate the expression of the catabolic enzyme ADAMTS5 and reduce the level of matrix protein ACAN [38,39], ultimately leading to the loss of cartilage matrix. Our results confirmed that BAY@OA-Der significantly inhibited NF-κB p65 phosphorylation, downregulated ADAMTS5 and restored ACAN expression at both mRNA and protein levels, thereby reversing the catabolic phenotype of chondrocytes and maintaining extracellular matrix homeostasis.

Reported NO-responsive nanocarriers generally exert only single-mode therapeutic functions [29]. Conventional NO-triggered delivery platforms only realize NO-stimulated drug release and possess no intrinsic chondroprotective effects, and their biological characterizations are limited to tumor cell lines rather than chondrocyte anti-osteoarthritis tests [26]. In addition, conventional NO-scavenging nanoparticles reported in most of the existing literature merely deplete excess NO, whose carrier backbones lack anti-chondrolytic activity and cannot synergistically block the NF-κB inflammatory cascade. Different from most artificially designed nanocarriers, the OA-based delivery system constructed in this study takes natural bioactive molecules as the building unit. This strategy has multiple merits: natural OA has good biocompatibility [40], so both blank OA-Der micelles and drug-loaded formulations show low cytotoxicity to chondrocytes; meanwhile, the integrated design of “carrier + therapeutic agent” abandons the single delivery function of traditional inert materials and realizes synergistic therapy [41,42]. Such carrier-free self-assembled supramolecular nanomedicines with dual therapeutic bioactivities have also been reported for tumor combination treatment [43]. It is worth noting that the self-assembly technology based on natural small molecules is simple and easy to operate [44], with mild preparation conditions [45], which lays a preliminary foundation for subsequent follow-up basic research. This work also proves that natural self-assembled molecules are promising building blocks for constructing stimulus-responsive nanocarriers for in vitro OA research, and the design idea can be extended to other natural active compounds with self-assembly properties.

Although the in vitro experiments have fully verified the performance and efficacy of BAY@OA-Der micelles, the current research still has certain limitations. This study only completed cell-level verification, and the actual performance of the nanosystem in the complex in vivo environment remains to be explored. The physiological environment in joints contains various proteins, enzymes and body fluids [46,47], which may affect the particle size, stability and responsive disassembly behavior of micelles [48,49]. In addition, the intra-articular retention time, in vivo biosafety, immune response and long-term toxicity of BAY@OA-Der also need to be systematically evaluated in animal osteoarthritis models [50]. Follow-up research will focus on establishing classical osteoarthritis animal models to further verify the in vivo anti-arthritic effect of the nanosystem and explore its in vivo distribution and metabolic characteristics.

## 5. Conclusions

In summary, this study successfully constructed NO-responsive drug-loaded micelles BAY@OA-Der based on the natural self-assembly characteristics of oleanolic acid. The nanosystem can respond to the high-NO microenvironment of inflammatory lesions to release drugs, synergistically inhibit the NF-κB pathway, protect chondrocytes and alleviate cartilage matrix degradation. This work not only provides a feasible strategy for osteoarthritis intervention in vitro, but also sets a reference for developing stimulus-responsive nanocarriers using natural self-assembled active molecules. Further systematic in vivo investigations are required to evaluate the practical application potential of this OA-derived self-assembled nanoplatform.

## Data Availability

The raw and processed experimental data supporting the findings of this study are available from the corresponding author upon reasonable request.

## Supplementary Materials

The following supporting information can be downloaded at https://www.mdpi.com/article/10.3390/bioengineering13080908/s1: Figure S1. ^13^C NMR characterization. Figure S2. Cell viability of chondrocytes treated with Control, DMSO and OA-Der, as determined by CCK-8 assay. Figure S3. Quantitative analysis of IL-6 concentration (pg/mL) in the culture supernatants of chondrocytes under different treatments: Control, LPS, LPS+BAY, LPS+OA-Der, and LPS+BAY@OA-Der. Figure S4. Quantitative analysis of TNF-α concentration (pg/mL) in the culture supernatants of chondrocytes under different treatments: Control, LPS, LPS+BAY, LPS+OA-Der, and LPS+BAY@OA-Der. Figure S5. Time-dependent ultraviolet changes of the compound after the introduction of nitric oxide.

## Abbreviations

The following abbreviations are used in this manuscript:

ACAN	Aggrecan
AFM	Atomic Force Microscopy
APC	Allophycocyanin
BAY@OA-Der	BAY 11-7082 loaded NO-responsive oleanolic acid derivative micelles
CCK-8	Cell Counting Kit-8
CDCl_3_	Deuterated chloroform
DMAP	4-Dimethylaminopyridine
DMEM/F12	Dulbecco’s Modified Eagle Medium/Nutrient Mixture F-12
DMSO-d_6_	Deuterated dimethyl sulfoxide
ESI-MS	Electrospray Ionization Mass Spectrometry
FBS	Fetal Bovine Serum
FTIR	Fourier Transform Infrared Spectroscopy
HAADF-STEM	High-Angle Annular Dark Field-Scanning Transmission Electron Microscopy
HRESI-MS	High-Resolution Electrospray Ionization Mass Spectrometry
LPS	Lipopolysaccharide
NF-κB	Nuclear Factor-κB
NO	Nitric oxide
OA	Oleanolic acid
OA-Der	NO-responsive oleanolic acid derivative
p-p65	Phosphorylated p65
PVDF	Polyvinylidene Fluoride
qPCR	Quantitative Real-Time Polymerase Chain Reaction
SDS-PAGE	Sodium Dodecyl Sulfate-Polyacrylamide Gel Electrophoresis
TEM	Transmission Electron Microscopy
UV-Vis	Ultraviolet–Visible Spectroscopy
β-actin	Beta-actin
ADAMTS5	A Disintegrin and Metalloproteinase with Thrombospondin Motifs
ROS	Reactive Oxygen Species
MMPs	Matrix Metalloproteinases
