# Supplementary material for: NO-Responsive Oleanolic Acid Self-Assembled Micelles Co-Loaded with BAY 11-7082 for Synergistic Chondroprotection and Anti-Osteoarthritis Therapy
Source: Bioengineering (Basel). 2026 Aug 11;13(8):908. doi: 10.3390/bioengineering13080908 (PMC13509297; doi:10.3390/bioengineering13080908)
Supplement: Supplementary file 1 [file bioengineering-13-00908-s001.zip › bioengineering-4460843-supplementary.pdf]

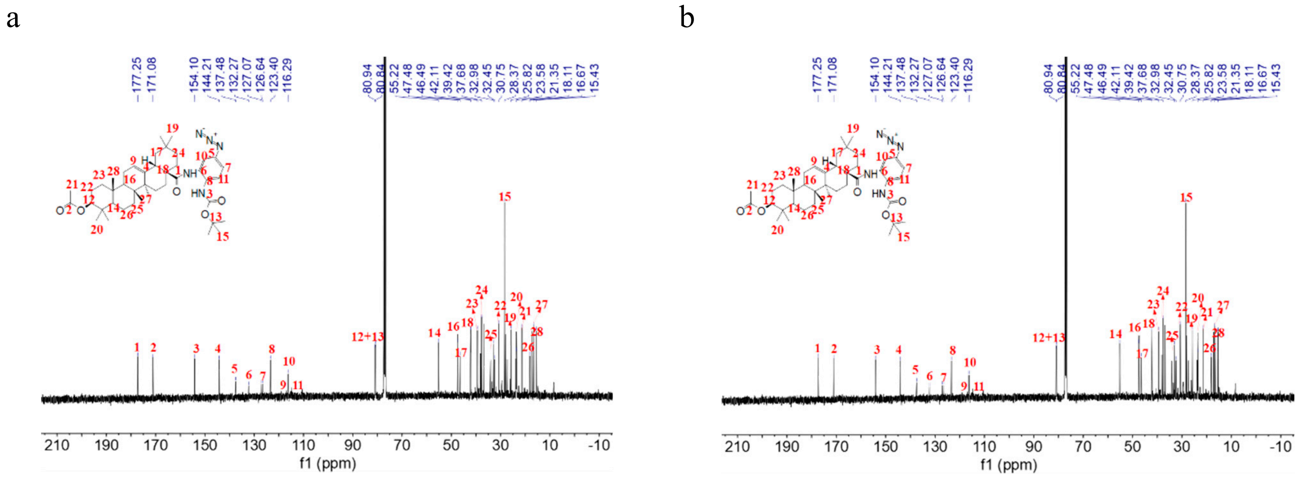

Figure S1. ^{13}C NMR characterization. (a) ^{13}C NMR spectrum of NO-responsive azide intermediate; (b) ^{13}C NMR spectrum of OA-Der.

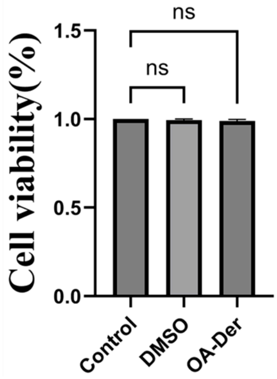

Figure S2. Cell viability of chondrocytes treated with Control, DMSO and OA-Der, as determined by CCK-8 assay.

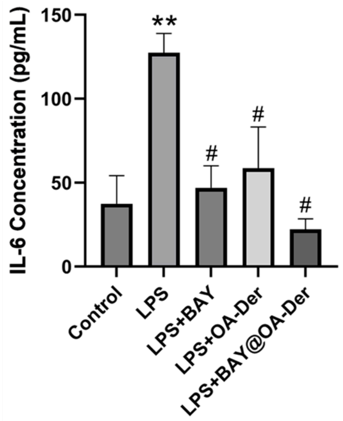

Figure S3. Quantitative analysis of IL-6 concentration (pg/mL) in the culture supernatants of chondrocytes under different treatments: Control, LPS, LPS+BAY, LPS+OA-Der, and LPS+BAY@OA-Der.

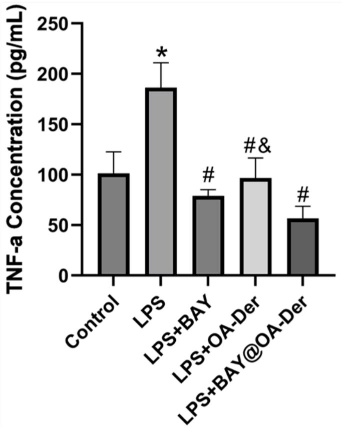

Figure S4. Quantitative analysis of TNF-α concentration (pg/mL) in the culture supernatants of chondrocytes under different treatments: Control, LPS, LPS+BAY, LPS+OA-Der, and LPS+BAY@OA-Der.

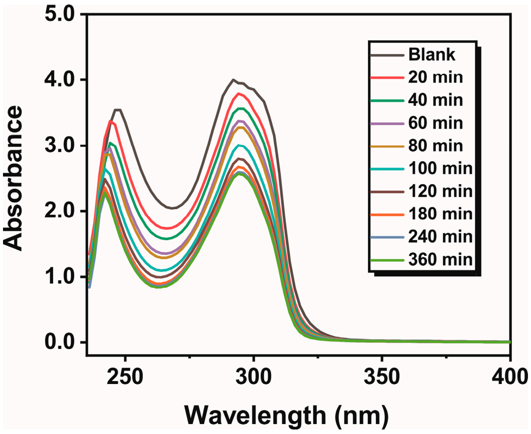

Figure S5. Time-dependent ultraviolet changes of the compound after the introduction of nitric oxide
